# Cancer-testis antigen KK-LC-1 is a potential biomarker associated with immune cell infiltration in lung adenocarcinoma

**DOI:** 10.1186/s12885-022-09930-5

**Published:** 2022-07-30

**Authors:** Yanli Kang, Yuhan Gan, Yingfeng Jiang, Jianbin You, Chen Huang, Qianshun Chen, Xunyu Xu, Falin Chen, Liangyuan Chen

**Affiliations:** 1 Department of Clinical Laboratory, Fujian Provincial hospital, Shengli Clinical Medical College of Fujian Medical University, No.134, East street, Gulou District, Fuzhou, 350001 China; 2 Department of Thoracic Surgery, Fujian Provincial hospital, Shengli Clinical Medical College of Fujian Medical University, No.134, East street, Gulou District, Fuzhou, 350001 China

**Keywords:** KK-LC-1, Lung adenocarcinoma, Cancer-testis antigen, Prognosis, Tumor immune infiltration

## Abstract

**Background:**

Cancer-testis antigens (CTAs) have emerged as potential clinical biomarkers targeting immunotherapy. KK-LC-1 is a member of CTAs, which has been demonstrated in a variety of tumors tissues and been found to elicit immune responses in cancer patients. However, the expression level and immune infiltration role of KK-LC-1 in lung adenocarcinoma (LUAD) remains to be elucidated.

**Methods:**

In this study, the mRNA expression and overall survival rate of *KK-LC-1* were evaluated by the TIMER and TCGA database in LUAD tissues and KK-LC-1 expression was further validated by clinical serum samples using quantitative RT-PCR. The relationship of KK-LC-1 with clinicopathologic parameters was analyzed. ROC curve result showed that miR-1825 was able to distinguish preoperative breast cancer patients from healthy people and postoperative patients. Then, the ROC curves were used to examine the ability of KK-LC-1 to distinguish preoperative LUAD patients from healthy and postoperative patients. The correlation between KK-LC-1 and infiltrating immune cells and immune marker sets was investigated via TIMER, TISIDB database, and CIBERSORT algorithm. The Kaplan-Meier plotter was used to further evaluate the prognostic value based on the expression levels of KK-LC-1 in related immune cells.

**Results:**

The results showed that KK-LC-1 was significantly over-expressed in LUAD, and high levels of expression of KK-LC-1 were also closely correlated with poor overall survival. We also found that KK-LC-1 associated with TMN stage, NSE and CEA. The ROC curve result showed that KK-LC-1 was able to distinguish preoperative LUAD cancer patients from healthy people and postoperative patients. Moreover, KK-LC-1 had a larger AUC with higher diagnostic sensitivity and specificity than CEA. Based on the TIMER, TISIDB database, and CIBERSORT algorithm, the expression of KK-LC-1 was negatively correlated with CD4+ T cell, Macrophage, and Dendritic Cell in LUAD. Moreover, Based on the TIMER database, KK-LC-1 expression had a remarkable correlation with the type markers of Monocyte, TAM, M1 Macrophage, and M2 Macrophage. Furthermore, KK-LC-1 expression influenced the prognosis of LUAD patients by directly affecting immune cell infiltration by the Kaplan-Meier plotter analysis.

**Conclusions:**

In conclusion, KK-LC-1 may serve as a promising diagnostic and prognostic biomarker in LUAD and correlate with immune infiltration and prognosis.

## Introduction

Lung cancer, the most common and fatal cancer in the world, causes more than 2.20 million new cases and 1.79 million patients die per year [1, 2]. Among all histological types, non-small cell lung cancer (NSCLC) makes up 80–85% of all lung cancer cases. Lung adenocarcinoma (LUAD) is the main member of NSCLC, which is the most invasive one [3]. Despite the conventional radiotherapy, chemotherapy and surgery have taken a leap forward, the five-year survival rate of NSCLC is still poor [4]. Recently, immunotherapy has emerged as an alternative treatment for lung cancer. For instance, pembrolizumab, an inhibitor of programmed death-1 (PD-1), has already been applied in the clinic [5]. However, the clinical results were still unsatisfactory because of drug resistance and adverse reactions [4]. Therefore, it is urgent to explore a reliable prognostic biomarker that can predict the prognosis of LUAD and improve the immunotherapy of patients.

Cancer-testis antigens (CTAs) may be suitable targets for cancer immunotherapy due to their immune-privileged properties. CTAs expressed restrictively in normal tissue except for testicular germ cells and various tumour types, including epithelial ovarian cancer, lung cancer, and cervical carcinoma [6–8]. Many reports found that CTAs were remarkedly correlated with the oncogenesis, metastasis, and unfavorable prognosis of tumors [9]. CTAs have strong immunogenicity and induce humoral immunity in several types of cancers [10, 11]. Kita-kyushu lung cancer antigen 1 (KK-LC-1), called CT83 or CXORF61, is a CTA that has epitope peptides recognised by cytotoxic T lymphocytes (CTLs) [12]. Fukuyama et al. first reported it in LUAD in 2006, which consisted of 556 base pair and located in chromosome Xq22 [12]. In recent years, the researches of KK-LC-1 mainly focused on the change of expression in different cancer tissues, especially gastric cancer, and the different therapeutic strategies such as photodynamic therapy and vaccine [13–17]. However, the expression level of KK-LC-1 in LUAD serum samples and the relationship between the expression level of KK-LC-1 with immune infiltration remain to be elucidated.

In this study, we analyzed the KK-LC-1 expression by using The Cancer Genome Atlas (TCGA) database. Quantitative real-time polymerase chain reaction (qRT-PCR) was used to further confirm KK-LC-1 expression in LUAD serum samples. Then, we explored the prognostic value of KK-LC-1 by plotting the survival curve and using the Kaplan-Meier plotter. In addition, we estimated the association between KK-LC-1 expression and tumor-infiltrating immune cells by using the Tumor Immune Estimation Resource (TIMER), CIBERSORT, and TISIDB database. Our results indicated that KK-LC-1 plays a non-redundant role in LUAD and is related to immune response.

## Materials and methods

### Clinical samples

The serum samples of 74 healthy individuals and 92 LUAD patients were obtained from Fujian Provincial Hospital (Fuzhou, China). All LUAD patients were diagnosed through histopathology without chemotherapy and radiotherapy before surgery. Then, we collected serum from 19 patients before and on the 5th day after surgery so that the serum samples were obtained preoperatively and postoperatively in pair. The clinicopathological data of all LUAD patients were recorded, including age, gender, tumor size, TNM stage etc. This study was approved by the Research Ethics Committee of Fujian Provincial Hospital (K-2021-040-04) and conformed to the ethical standards of the 1964 Helsinki Declaration, and all subjects and voluntarily signed informed consent forms.

### Quantitative real-time polymerase chain reaction (qRT-PCR)

We first extracted total RNA from serum samples according to TRIzolTM LS Reagent instructions. Then, the RNA was reversely transcribed into cDNA under 37 °C for 15 min, followed by 85 °C for 5 s. CDNA was finally amplified by TB Green® Premix Ex Taq™ II Kit (RR820A; TAKARA, Tokyo, Japan) manufacturer’s instruction in 40 cycles of denaturation at 95 °C for 10 min, followed by 95 °C for 15 s, with extension at 60 °C for 1 min using LightCycler 480 System. qRT-PCR’s primer sequences were as follows:

- GAPDH,Forward:GGCCTCCAAGGAGTAAGACC, Reverse:AAGGGGAGATTCAGTGTGGTG;
- KK-LC-1,Forward:ATGAACTTCTATTTACTCCTAGCGAGC,
- Reverse: CTACAATATTGAGTGTGGGAAATTATTTAA.

The relative expression levels of KK-LC-1 was calculated by 2^-ΔΔ^CT method.

### TIMER database

TIMER database (https://cistrome.shinyapps.io/timer/) is a comprehensive online tool, which can not only systematically evaluate immune infiltrates of different cancers, but also compare the differential expression between cancer and normal tissue [18]. In present study, we used the TIMER database to explore the difference KK-LC-1 expression between various cancers and adjacent normal samples. Then, we analyzed the relationship between KK-LC-1 and immune cell infiltration in LUAD. Moreover, the relationship between immune cell infiltration and corresponding gene markers was analyzed.

### TCGA database

The gene expression profiles were downloaded from the TCGA database (http://portal.gdc.cancer.gov/). We analyzed the expression of *KK-LC-1* mRNA between LUAD and adjacent para-cancerous lung tissues. In addition, the association between LUAD and matched normal tissues was also further validated by TCGA.

### Kaplan-Meier potter analysis

The association between 54,675 genes expression and survival from 21 tumor types can be assessed by Kaplan-Meier potter (http://kmplot.com) [19]. We analyzed the prognosis value of KK-LC-1 expression in LUAD. Besides, prognosis of KK-LC-1 expression based on various immune cell were evaluated by Kaplan–Meier plotter. Log-rank *P*-values (*P* < 0.05) and hazard ratio (HR) with 95% confidence intervals were computed.

### TISIDB database analysis

The TISIDB (http://cis.hku.hk/TISIDB) database is high-throughput screening techniques, molecular profiling, and para-cancerous multiomic data, as well as various resources for immunological data obtained from seven public databases [20]. TISIDB enables analysis of associations between KK-LC-1 and tumor-infiltrating immune cells.

### CIBERSORT algorithm

CIBERSORT (https://www.biostars.org/p/428905/) is an analytical tool, which aids in evaluating the abundances of member cell types in a mixed cell population through gene expression data [21]. We explored the relationship between high and low expression of KK-LC-1 and 22 types of tumor-infiltrating immune cells using CIBERSORT algorithm.

### Statistical analysis

R software package was used to analyze TCGA data after download. Statistical analysis was processed by the Statistical Program for Social Sciences (SPSS) 22.0 software (SPSS, Chicago, IL) and GraphPad Prism 5.0 (GraphPad Software, La Jolla, CA). The qRT-PCR data were analyzed using the unpaired Student’s t-test or paired t-test. The Receiver operating characteristic (ROC) curve, and the area under the curve (AUC) were performed to evaluate the diagnostic value of KK-LC-1. The Kaplan-Meier plotter was employed to generate survival curves. Gene expression corrections were performed in the TIMER databases by Spearman’s correlation analysis with the hazard ratio (HR) and *P*-values or Cox *P*-values. *P* < 0.05 was statistically significant.

## Results

### The mRNA expression of *KK-LC-1* in pan-carcinoma and associated with the prognosis of LUAD

**Fig. 1:**
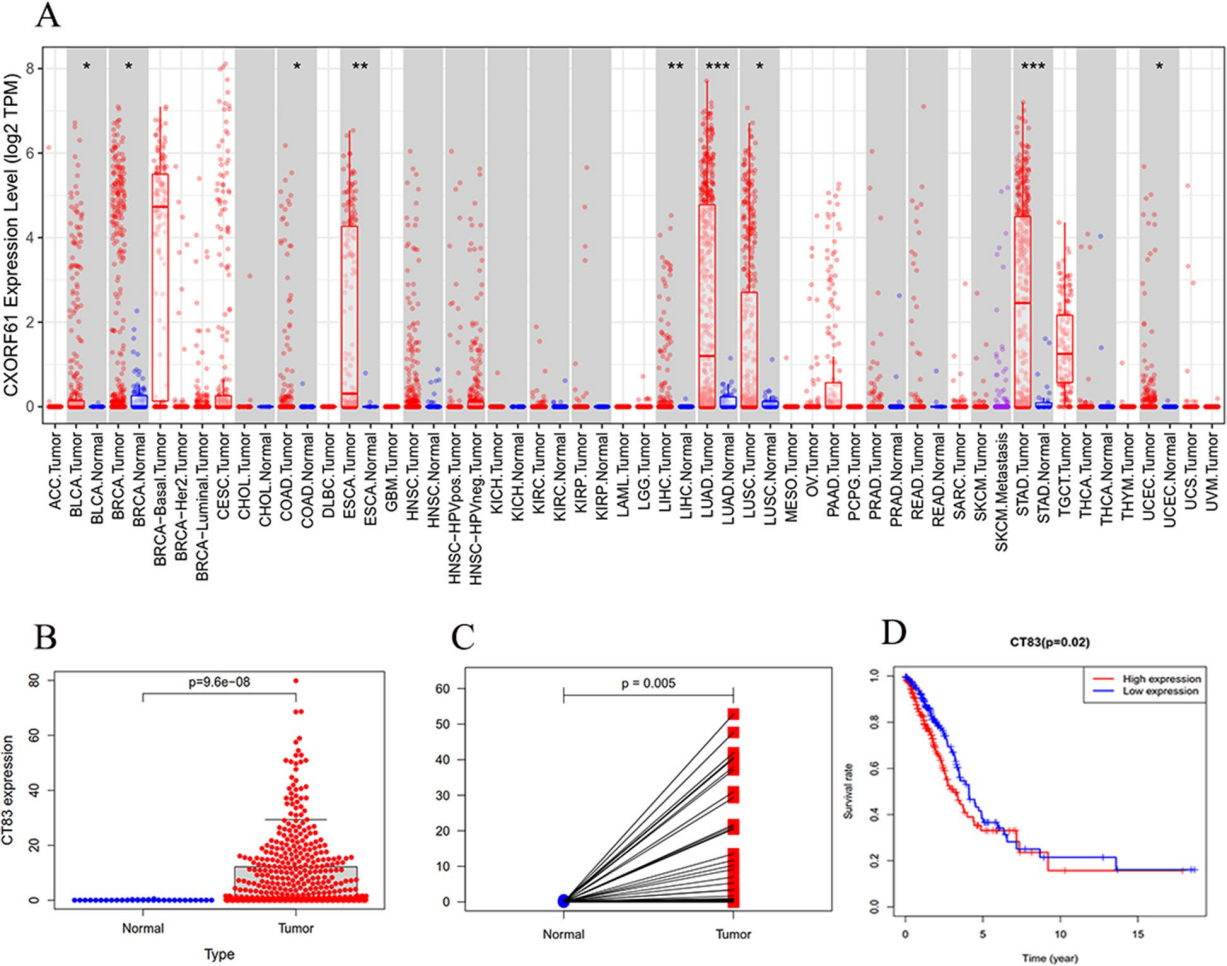
KK-LC-1 expression in various human cancers and related to prognosis in LUAD. **A** Human KK-LC-1 expression in various tumors according to the TIMER database. **B** The expression levels of KK-LC-1 in LUAD and para-cancerous lung tissues. **C** KK-LC-1 expression in LUAD and matched normal tissues by TCGA database. **D** The overall survival rate of KK-LC-1 expression in LUAD

We firstly analyzed KK-LC-1 expression at the mRNA level, and then explored its prognosis value in patients with LUAD. The differential mRNA expression of *KK-LC-1* between diverse tumor tissues and matched para-cancerous tissues was analyzed by TIMER database. KK-LC-1 expression was significantly higher in tumor tissue than matched para-cancerous tissues, including LUAD (Fig. 1A). In a dataset from TCGA, the expression level of KK-LC-1 was remarkably increased in 538 LUAD tissues compared with 59 normal tissues (Fig. 1B), and the result was consistent with the 59 matched tissue samples from the LUAD patients (Fig. 1C). In addition, we performed a survival analysis in LUAD, which revealed that the higher expression of KK-LC-1 was correlated with a poorer prognosis (Fig. 1D).

### The mRNA expression of *KK-LC-1* in serum and associated with the diagnosis of LUAD

**Fig. 2:**
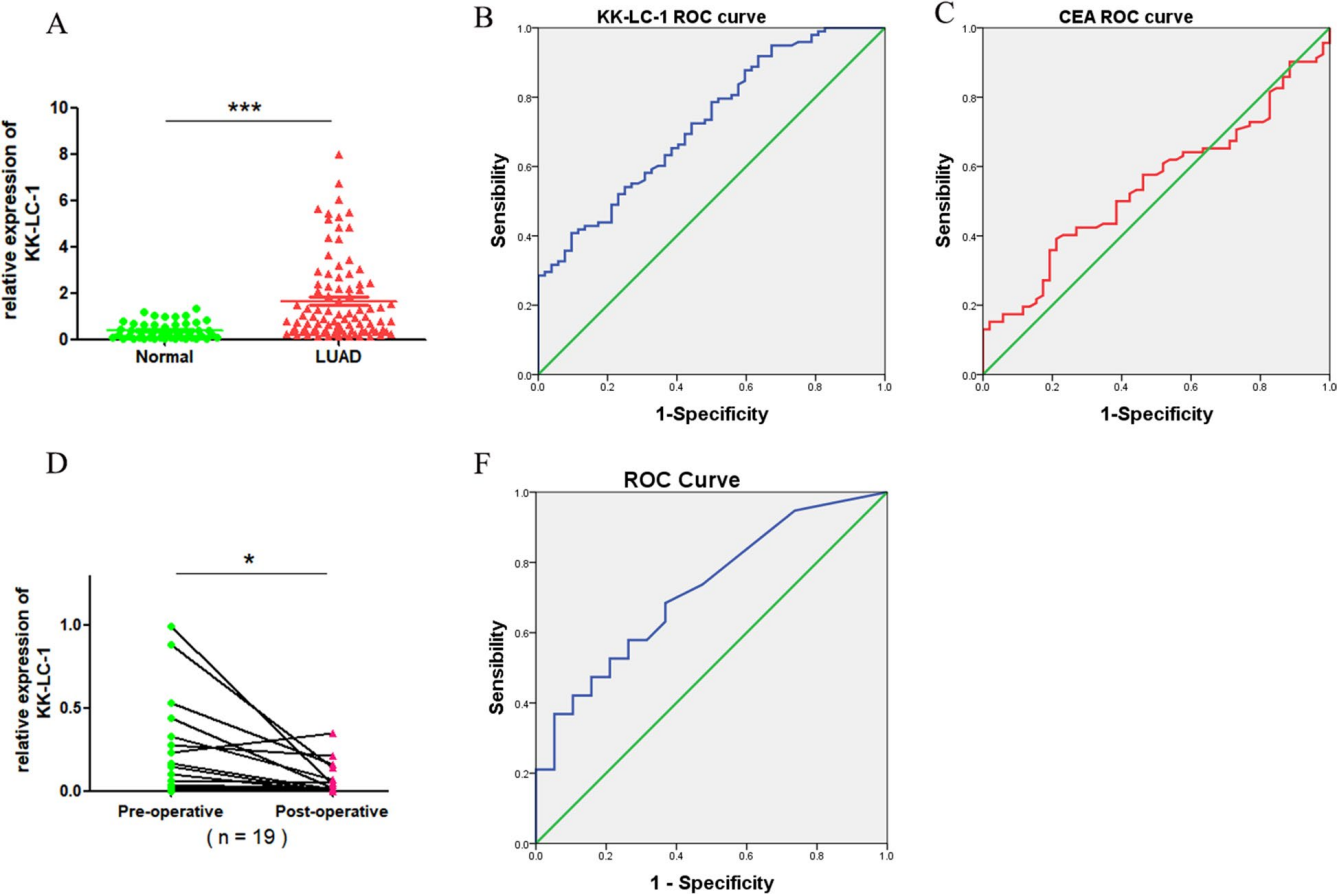
KK-LC-1 expression in serum and related to diagnosis in LUAD. **A** The expression level of KK-LC-1in LUAD serum and healthy person’s serum. ROC curve of serum **B** KK-LC-1 and **C** CEA. **D** KK-LC-1 expression in LUAD patient’s preoperative and postoperative serum. **E** ROC curve of serum KK-LC-1 to validate pre-operative cases from post-operative in LUAD

Next, we evaluated the diagnostic significance of KK-LC-1 in the serum of LUAD patients. The expression level of KK-LC-1 in 92 LUAD patient’s serum was higher than 47 healthy control by qRT-PCR (Fig. 2A). Then, the relationship between the clinicopathological features of LUAD samples and the expression level of KK-LC-1 was analyzed. As shown in Table 1, KK-LC-1 expression was correlated with the stage, Carcino-Embryonic Antigen (CEA), and Neuron-Specific Enolase (NSE) (*P* < 0.05). The receiver operating characteristic (ROC) curves and the area under curve (AUC) analyses were performed for the diagnostic role of KK-LC-1. Compared with CEA, KK-LC-1 had a higher AUC (0.794 VS. 0.565) (Fig. 2B, C). Interestingly, the expression level of KK-LC-1 in 19 LUAD patients’ serum was significantly decreased than that in patients after operation (Fig. 2D), and KK-LC-1 had a good ability to discriminate distinguish preoperative LUAD cancer patients from and postoperative patients with an AUC of 0.720 (Fig. 2E).

**Table 1:** Relationship of KK-LC-1 expression and clinicopathologic parameters

Clinicopathological factor		KK-LC-1	
	n	Mean ± SEM	P
Age (yr)			0.5696
< 60	60	1.571 ± 0.2253	
≥ 60	32	1.793 ± 0.3247	
Gender			0.6368
Male	33	1.766 ± 0.3441	
Female	59	1.583 ± 0.2159	
Smok			0.7261
Yes	7	1.405 ± 0.5871	
No	78	1.655 ± 0.2057	
Diameter			0.175
< 3	72	1.627 ± 0.1947	
≥ 3	10	2.472 ± 0.9061	
Stage			**0.0087**
I	65	1.340 ± 0.1941	
II + III + IV	27	2.392 ± 0.3912	
CEA			**0.0416**
<5 ng/ml	75	1.476 ± 0.1855	
≥ 5 ng/ml	13	2.548 ± 0.6442	
NSE			**0.0225**
<16.3 ng/ml	41	1.439 ± 0.2629	
≥ 16.3 ng/ml	19	2.641 ± 0.4978	

### Association between KK-LC-1 expression and infiltration levels of immune cells in LUAD

**Fig. 3:**
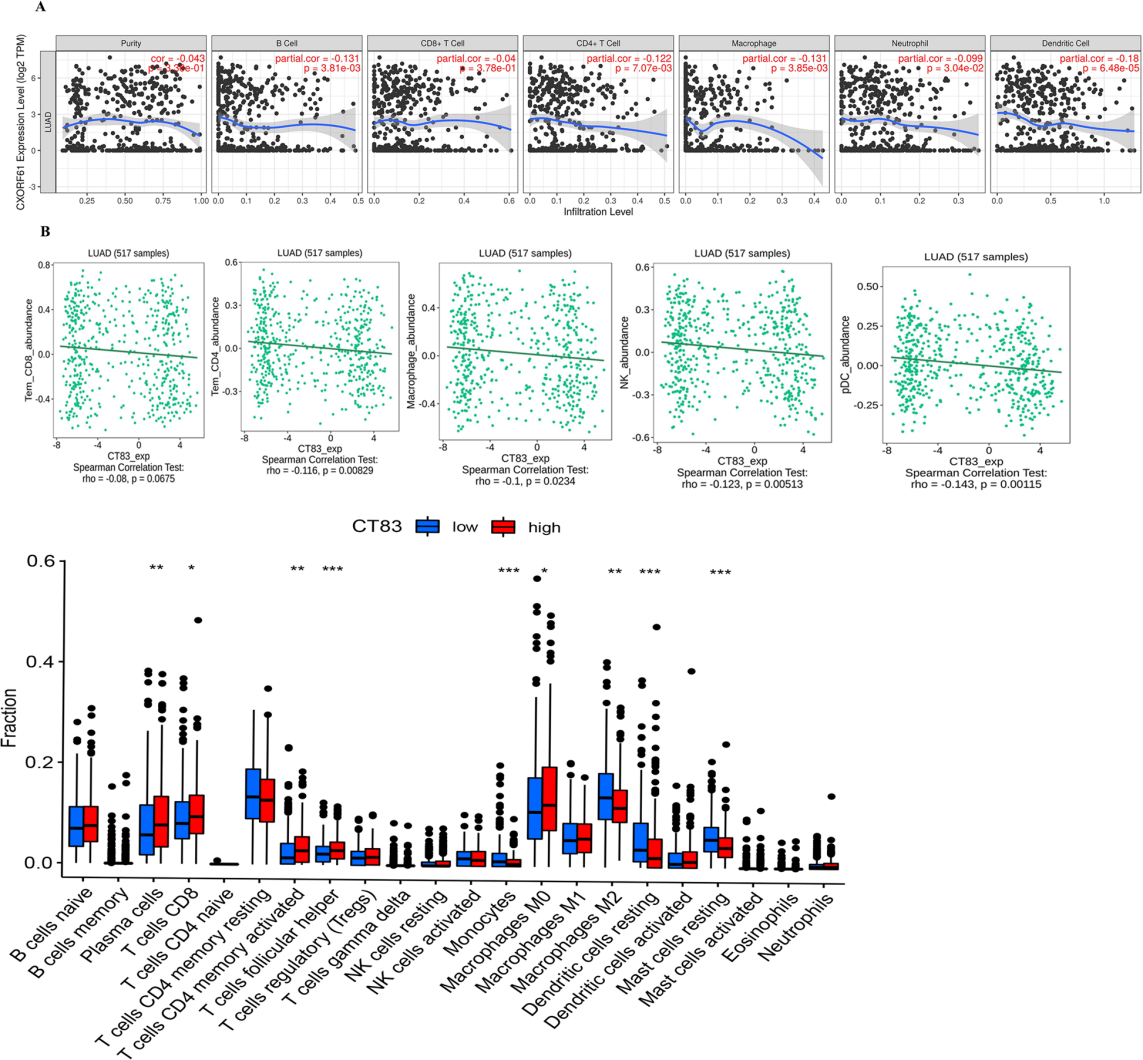
Association of KK-LC-1 expression with immune infiltration in LUAD according to the TIMER database. **A** The correlation between KKLC and immune cells by TIMER. **B** The correlation between KKLC and immune cells by TISIDB. **C** 22 types of tumor-infiltrating immune cells were analyzed in high and low KK-LC-1 expression groups

We then evaluated the correlation of KK-LC-1 expression with the infiltration levels of immune cells in LUAD based on TIMER database. As shown in Fig. 3A, the expression level of KK-LC-1 was significantly negatively correlated with B cell (*r* = − 0.131, *p* = 3.81e–03), CD4+ T cell (*r* = − 0.122, *p* = 7.07e–03), macrophage (*r* = − 0.131, *p* = 3.85e–03), neutrophil (*r* = − 0.099, *p* = 3.04e–02), and dendritic cell (*r* = − 0.18, *p* = 6.48e–05). However, there was no significant correlation between KK-LC-1 and tumor purity (*r* = − 0.043, *p* = 3.36e–01) and CD8+ T cell (*r* = − 0.04, *p* = 3.78e–01) (Fig. 3A). This negative correlation between the expression level of KK-LC-1 and CD4+ T cell (*r* = − 0.116, *p* = 8.29e–03), macrophage (*r* = − 0.1, *p* = 2.34e–02), Natural killer cell (*r* = − 0.123, *p* = 5.13e–03), and dendritic cell (*r* = − 0.143, *p* = 1.15e–03) was found in TISIDB database (Fig. 3B). Next, we further explored the difference between infiltrating immune cells and KK-LC-1 expression by CIBERSORT. The results indicated the higher KK-LC-1 expression related to the higher immune cells infiltration in plasma cell, activated CD4+ memory T cell, follicular helper T cell, macrophages M0, while monocyte, macrophages M2, resting dendritic cell, resting mast cell had opposite result (Fig. 3C).

### Association between KK-LC-1 and immune cell type markers

**Fig. 4:**
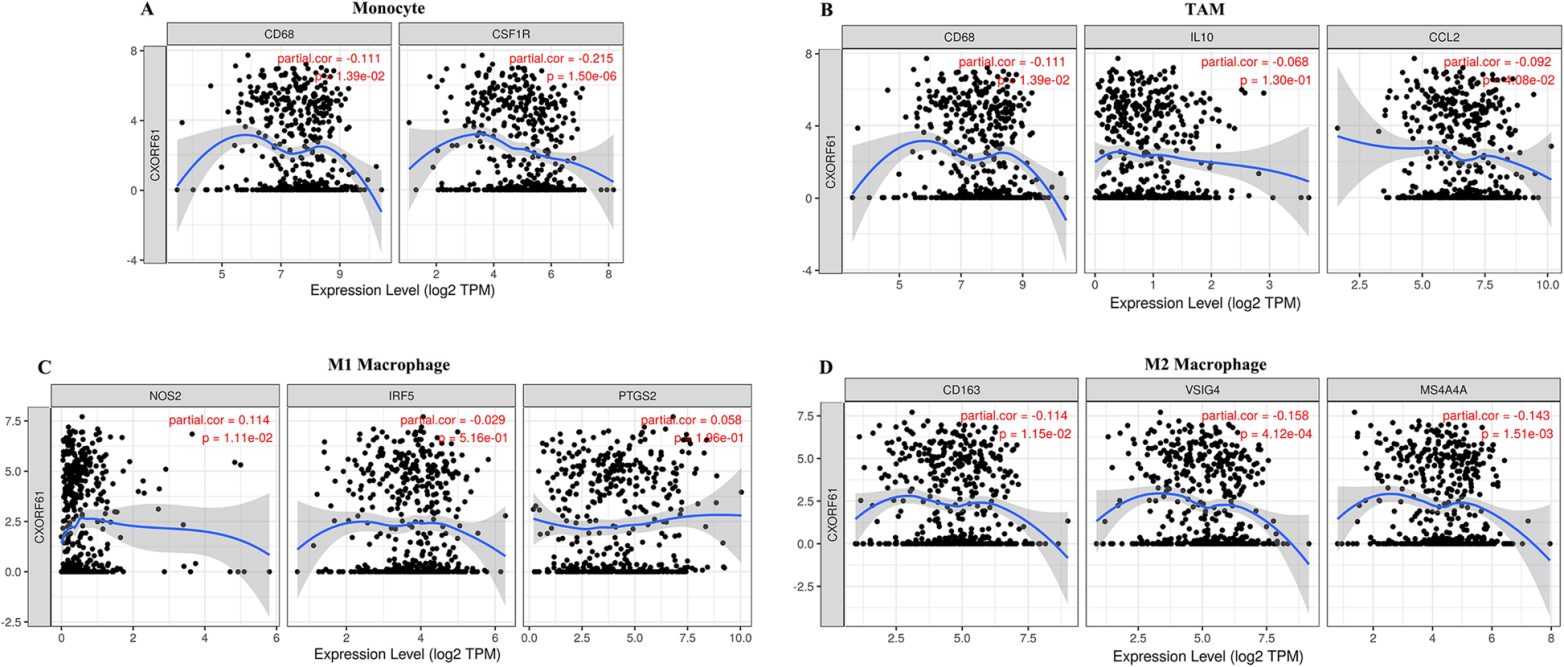
Association of KK-LC-1 expression with immune marker sets in LUAD based on TIMER database. The correlation between KK-LC-1 expression and the gene markers of **A** Monocyte (CD68 and CSF1R); **B** TAM (CD68, IL10 and CCL2); **C** M1 Macrophage (NOS2, IRF5 and PTGS2); **D** M2 Macrophage (CD163,VSIG4 and MS4A4A)

TIMER was used to further investigate the correlation between KK-LC-1 and immune cell markers. After the adjustment for purity, we focused on the immune cell markers of B Cell, T Cell, CD8 + T Cell, Monocyte, M1 Macrophage, M2 Macrophage, TAM, Neutrophil, Natural killer cell, Dendritic cell, and functional T cell markers of Th1, Th2, Tfh, Th17, Treg, T cell exhaustion (Table 2). As shown in Table 2 and Fig. 4, KK-LC-1 expression had a remarkable correlation with CD68 and CSF1R of Monocyte (*P* < 0.05), CD68 and CCL2 of TAM (*P* < 0.05), NOS2 of M1 Macrophage (*P* < 0.05), CD163, VSIG4 and MS4A4A of M2 Macrophage (*P* < 0.05). Taken together, KK-LC-1 may be participated in the LUAD immune response by regulating the immune cells.

**Table 2:** Relationship of KK-LC-1 with immune cell type markers by TIMER database

Description	Marker genes	None Cor	P	Purity Cor	P
B Cell	CD19	0.097	*	0.088	0.052
	CD79A	0.095	*	0.079	0.081
T Cell **(general)**	CD3D	0.019	0.672	−0.016	0.722
	CD3E	−0.013	0.761	−0.054	0.229
	CD2	−0.037	0.403	− 0.082	0.068
CD8 + T Cell	CD8A	0.04	0.36	0.015	0.738
	CD8B	0.03	0.501	0.009	0.837
	CD45(PTPRC)	−0.099	*	−0.146	**
Monocyte	CD86	−0.083	0.061	−0.129	**
	CD115(CSF1R)	−0.171	***	−0.215	***
M1 Macrophage	INOS (NOS2)	0.123	**	0.114	*
	IRF5	−0.016	0.721	−20.29	0.516
	COX2(PTGS2)	0.065	0.14	0.058	0.196
M2 Macrophage	CD163	−0.08	0.069	−0.114	*
	VSIG4	−0.126	**	−0.158	***
	MS4A4A	−0.107	*	−0.143	**
TAM	CD68	−0.08	0.071	−0.111	*
	IL10	−0.052	0.243	−0.068	0.13
	CCL2	−0.073	0.097	−0.092	*
Neutrophil	CD66b (CEACAM8)	−0.169	***	−0.186	***
	CD15(FUT4)	0.094	*	0.101	*
	CCR7	−0.03	0.493	−0.061	0.173
Natural killer cell	KIR2DL1	0.008	0.849	−0.008	0.854
	KIR2DL3	0.034	0.447	0.026	0.0572
	KIR2DL4	0.142	**	0.135	**
	KIR3DL1	0.087	*	0.089	*
	KIR3DL2	0.047	0.288	0.045	0.318
	KIR3DL3	0.116	0.087	0.116	**
	KIR2DS4	0.024	0.585	0.022	0.623
Dendritic cell	BDCA-1 (CD1C)	−0.214	***	−0.238	***
	BDCA-3 (THBD)	−0.16	***	−0.175	***
	BDCA-4 (NRP1)	−0.032	0.472	−0.036	0.426
	CD11c (ITGAX)	−0.053	0.227	−0.088	0.051
Th1	T-bet (TBX21)	−0.024	0.582	−0.047	0.293
	STAT4	−0.014	0.752	−0.046	0.309
	TNF-α (TNF)	−0.061	0.169	−0.084	0.062
	STAT1	0.065	0.143	0.046	0.306
Th2	GATA3	−0.031	0.483	−0.073	0.105
	STAT5A	−0.122	**	−0.152	***
	STAT6	−0.027	0.534	−0.033	0.465
Tfh	BCL6	0.021	0.641	0.017	0.714
	IL21	0.047	0.292	0.021	0.641
Th17	STAT3	0.029	0.516	0.03	0.454
	IL17A	0.055	0.214	0.05	0.266
Treg	FOXP3	0.015	0.738	−0.025	0.582
	CD25(IL2RA)	−0.026	0.553	−0.059	0.195
	CCR8	0.007	0.882	−0.082	0.542
	TGFβ (TGFB1)	−0.064	0.167	−0.075	0.095
	STAT5B	−0.021	0.639	−0.01	0.828
T cell exhaustion	PD-1 (PDCD1)	0.021	0.633	−0.001	0.986
	CTLA4	0.009	0.839	−0.023	0.603
	LAG3	0.013	0.763	−0.003	0.940
	TIM-3 (HAVCR2)	−0.088	*	−0.139	**
	GZMB	0.13	**	0.114	*

Cor, R value of Spearman’s correlation; None, correlation without adjustment;Purity,correlation adjusted by purity; *TAM* tumor-associated macrophage; *Th* T helper cell; *Tfh* Follicular helper T cell; *Treg* regulatory T cell. (∗*p* < 0.01, ∗∗*p* < 0.001, ∗∗∗*p* < 0.0001)

### Prognostic value analysis of KK-LC-1 based on immune cells in LUAD

**Fig. 5:**
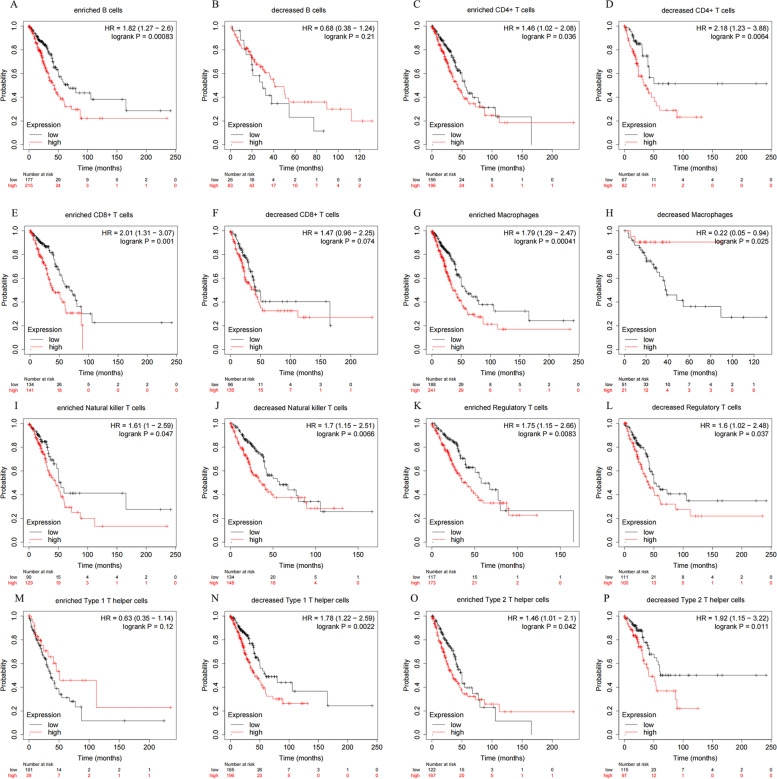
The prognostic value of high and low expression of KK-LC-1 in LUAD according to immune cell subgroups using Kaplan-Meier potter. The increased and decreased expression of KK-LC-1 in various immune cell subgroups have diverse prognosis in LUAD (**A**-**H**)

Finally, we performed prognostic analyses based on KK-LC-1 expression in LUAD in different immune cell subgroups. High expression of KK-LC-1 in decreased macrophages cohort in LUAD was associated with better prognosis (Fig. 5H). Meanwhile, high expression of KK-LC-1 in enriched B cells, enriched CD8+ T cells, enriched macrophages, enriched/decreased CD4+ T cells, enriched/decreased natural killer T cells, enriched/decreased regulatory T cells, decreased type 1 T helper cells and enriched/decreased type 2 T helper cells cohorts in LUAD was associated with poor prognosis (Fig. 5A, C-E, G, I-L, N-P). Besides, there was no significant association between low/high KK-LC-1 expression and LUAD patient prognosis in decreased B cells and decreased CD8+ T cells, enriched type 1 T helper cells cohorts (Fig. 5B, F, M). The result suggested that KK-LC-1 expression influenced the prognosis of LUAD patients by directly affecting immune cell infiltration.

## Discussion

CTAs referred to as CT antigen is expressed only in testis and embryonic primordial cells, but they can also be abnormally expressed in malignant tumors. Previous researches showed that CTAs were involved in the occurrence and development of tumors [22]. CTA can be used as an immunotherapy target mediated by cytotoxicity T lymphocyte [23]. Some CTAs were identified to have immunogenicity, indicating the possibility of tumor immunotherapy targets [24–26]. In previous studies, compared with NY-ESO-1 (10.5%), which has been used in clinical immunotherapy, KK-LC-1 (32.6%) has a higher expression level in NSCLC [27]. KK-LC-1 also has been reported that be highly expressed in lung cancer, gastric cancer, triple-negative breast cancer (TNBC), and hepatocellular carcinoma (HCC) [12, 13, 28, 29]. In this study, we determined the database-derived tissues and clinic-derived serum expression levels of KK-LC-1 in LUAD patients, and analyzed the correlation of KK-LC-1 with immune cell infiltration of LUAD.

When KK-LC-1 was first discovered, it was positively expressed in 50% of 15 lung cancer cell lines and 38% of LUAD tissues [12]. Hsu et al. reported that KK-LC-1 expression level was higher in LUAD than LUSC [30]. The expression of KK-LC-1 was intimately related to tumor stage and lymph node metastasis in lung cancer patients [31]. Our results from TIMER and TCGA databases showed that KK-LC-1 expression was up-regulated in LUAD tissues compared with normal tissues. Meanwhile, high levels of expression of KK-LC-1 were also closely correlated with poor overall survival, suggesting that KK-LC-1 may be a promising biomarker for survival prediction in LUAD. The above results only focus on the expression of KK-LC in tissue and cells but not in serum or plasma. As we all known, compared with tissue samples, serum has the advantages of being less invasive, easy to obtain and repeatable. In our study, it was the first time to analyze KK-LC-1 expression in serum from LUAD patients. As expected, serum KK-LC-1 expression was elevated in serum from LUAD patients, and was significantly correlated with TNM stage, CEA and NSE levels. In addition, serum KK-LC-1 could be used to differentiate LUAD patients from healthy controls. Moreover, serum KK-LC-1 expression was remarkably decreased after surgery, indicating serum KK-LC-1 was closely correlated with tumor occupying. These findings implied that serum KK-LC-1 could serve as a tumor marker for a diagnostic and prognostic predictor in LUAD patients.

Paret et al. demonstrated that KK-LC-1 could induce a strong antigen-specific immune response in TNBC based on specific recognition of TCR epitopes through vitro and vivo experiments [32]. Marcinkowski et al. also reported that KK-LC-1 has great potential in T cell receptor (TCR) gene-engineered T cells therapy for gastric cancer [33]. These evidences suggested that KK-LC-1 might be a progressing immunotherapy target. In our study, we found that KK-LC-1 was negative correlated with immune cell infiltration CD4+ T cell, macrophage, neutrophil and dendritic cell, which was further validated by immune cell surface markers. It is well known that CD4+ T cell, macrophage, neutrophil and dendritic cell play antitumor roles in cancers [34–36]. The negative correlation between KK-LC-1 and immune cells further indicated that KK-LC-1 seemed to play a important role in promoting cancer. At the same time, KK-LC-1 directly affected patient outcomes by influencing immune cell infiltration, which was similar with the research by Yoshinobu Ichiki et al. in LUSC [14]. Hsu et al. also reported that KK-LC-1 was related to the abundance of macrophages and CD4 + T cells through QuantiSeq algorithm in lung cancer [30]. Our results showed that KK-LC-1 expression level was strongly correlated with lung cancer-related immune cells infiltration, and KK-LC-1 affected the prognosis of LUAD by modulating the infiltration level of tumor infiltrating immune cells (TIICs).

However, the research on relevant mechanisms is limited in our study. In fact, to date, there are few reports on the mechanism of KK-LC-1 in human malignant tumors. Methylated CpG islands associated with the CT genes in normal somatic cells become demethylated in cancer cells, indicating activation of their expression. Xie et al. reported that PIWIL1 is considered to be a highly expressed CT gene in LUAD, and promoter DNA hypomethylation of PIWIL1 could contribute to its aberrant expression in LUAD [37]. KK-LC-1 expression is activated by treatment with the hypomethylating agent 5-aza-2′-deoxycytidine in KK-LC-1 negative breast cancer cell lines [15, 32].

To sum up, the high expression level of serum KK-LC-1 in patients with LUAD was closely related to TNM stage, CEA and NSE. The expression of serum *KK-LC-1* mRNA decreased significantly after surgery. KK-LC-1 expression has a strong correlation with lung cancer-related immune cells infiltration, which can affect the prognosis of LUAD. Our findings suggest that KK-LC-1 played a non-redundant role in tumor immunology and served as a diagnostic biomarker in LUAD. However, it needs a further study to verify the above results with large sample size, and to explore the mechanisms and immunoregulatory functions of KK-LC-1 in LUAD.

## Conclusions

KK-LC-1 may serve as a promising diagnostic and prognostic biomarker in LUAD and is corelated with immune infiltration and prognosis.

## Data Availability

The data, which was used in this study can be found at the following websites and thoracic surgery, Fujian Provincial Hospital. All methods were carried out in accordance with relevant guidelines and regulations. The differentially expressed in Pan-cancer was analyzed by TIMER (https://cistrome.shinyapps.io/timer/) and TCGA databases (http://portal.gdc.cancer.gov/). The Kaplan-Meier potter (http://kmplot.com) was utilized to evaluated prognosis. The association between KK-LC-1 and immune infiltration was explored by TIMER and TISIDB database (http://cis.hku.hk/TISIDB), and CIBERSORT (https://www.biostars.org/p/428905/) algorithm. The datasets used during the current study are available from the corresponding author on reasonable request.

## Abbreviations

CTAs: Cancer-testis antigens
KK-LC-1: Kita-kyushu lung cancer antigen 1
NY-ESO-1: New York esophageal squamous cell carcinoma 1
NSCLC: Non-small cell lung cancer
LUAD: Lung adenocarcinoma
LUSC: Lung squamous cell carcinoma
BRCA: Breast invasive carcinoma
ESCA: Esophageal carcinoma
PAAD: Pancreatic adenocarcinoma
STAD: Stomach adenocarcinoma
TGCT: Testicular germ cell tumors
TNBC: Triple negative breast cancer
HCC: Hepatocellular carcinoma
PD-1: Programmed death-1
CEA: Carcino-Embryonic Antigen
NSE: Neuron-Specific Enolase
CTL: Cytotoxic T lymphocytes
TCR: T Cell receptor
qRT-PCR: Quantitative real-time polymerase chain reaction
TIICs: Tumor infiltrating immune cells
TCGA: The Cancer Genome Atlas
TIMER: Tumor Immune Estimation Resource
TISIDB: An Integrated Repository Portal For Tumor-Immune System Interactions
ROC: Receiver operating characteristic
AUC: Area under the curve
OS: Overall survival
HR: Hazard Ratio
SPSS: Statistical Program for Social Sciences
